# Clinical Audit as a Quality Improvement Tool in Measurements of Lying and Standing Blood Pressure for Elderly Patients Admitted With a Hip Fracture

**DOI:** 10.7759/cureus.16781

**Published:** 2021-07-31

**Authors:** Isaac C Okereke, Kingsley Mmerem, Mohamed Aly

**Affiliations:** 1 Trauma and Orthopaedics, The Royal London Hospital, London, GBR

**Keywords:** orthostatic hypotension, quality improvement, clinical audit, staff education, blood pressure

## Abstract

Background: Around one in three adults aged 65 years and over will have a fall at home within a one-year period. Falls are estimated to cost the NHS more than £2.3 billion per year. The National Institute for Health & Care Excellence (NICE) guidelines recommend older people who present for medical attention because of a fall, report recurrent falls in the past year, or demonstrate abnormalities of gait and/or balance should be offered a multifactorial falls risk assessment which includes a cardiovascular examination and review of medications. Orthostatic hypotension (OH) is a common cardiovascular disorder, independently associated with an increased risk of falls in the elderly.

Aims & Objectives: This study was carried out to assess improvement in lying and standing blood pressure (LSBP) measurement using clinical audit and staff education.

Method: An initial audit of patients over the age of 60, admitted with a hip fracture between the 14th of April and the 25th of May 2020 to assess measurement and accurate recording of LSBP. This cycle was followed by brainstorming, root cause analysis, teaching sessions for staff, and use of aide-memoires. A second audit cycle of patients was admitted with a hip fracture secondary to a fall between the 10th of August and the 21st of September 2020.

Result: Our initial audit results showed 68% of patients who met the criteria in the NICE guidelines on measurement of LSBP were not being assessed for OH. Following interventions, the second audit cycle showed significant improvement in compliance, confirming audits to be a powerful tool in quality improvement programs.

## Introduction

A fall is defined as an event that results in a person coming to rest inadvertently on the ground or other lower level [1]. Whilst not all falls are fatal, most falls cause significant injuries such as fragility fractures, functional impairment, longstanding pain, and intracranial bleeds in predisposed individuals.

The UK government's Public Health Outcomes Framework reports the rates of emergency hospital admissions due to falls in patients 65 years and over in the financial year 2018-2019 as 2,198 per 1000,000; an equivalent to 226,567 hospital admissions [2]. Around one in three adults aged 65 and over will have a fall at home within one year. The rate of falls increases dramatically in both sexes and within all races and ethnicities as people age [3]. The human cost of falling is unquantifiable and includes physical distress, pain, injury, loss of confidence, loss of independence and mortality. Falls are a public health concern estimated to cost the NHS more than £2.3 billion per year and have a negative impact on patients' quality of life [4]. Risk factors for falls include age, female gender, visual impairments, gait imbalance polypharmacy, and previous falls history.

In elderly patients, a fall may indicate an underlying medical problem such as a lower respiratory tract infection, a urinary tract infection, an acute exacerbation of chronic disease, or it may be due to the existence of orthostatic hypotension [5]. Up to 30% of falls can be prevented [6] by using a standardized multidisciplinary approach. The National Institute for Health & Care Excellence (NICE) guidelines recommend older people who present for medical attention because of a fall, report recurrent falls in the past year, or demonstrate abnormalities of gait and balance be offered a multifactorial falls risk assessment which includes a cardiovascular examination and a review of their current medications by managing clinicians [7].

Orthostatic hypotension (OH) is a common cardiovascular disorder in the elderly population due to age-related autonomic dysfunction. It can present with or without signs of neurodegenerative disease and occurs due to a blunted response by the neurogenic adaptive mechanisms that recruit the sympathetic system to compensate for venous filling that ensues when standing upright from a recumbent position [8,9]. The prevalence of OH in the general population is about 6% and rises exponentially with age to a range of between 10% to 30% in older adults [10-13]. Risk factors for OH include age, adrenal insufficiency, hypertension and use of antihypertensive drugs, smoking status, low body mass index (BMI), and diabetes [14,15]. OH is assessed by taking blood pressure measurements in the supine and upright positions (lying and standing blood pressure [LSBP]) and is confirmed by a persistent systolic/diastolic blood pressure drop >20/10mmHg within three minutes of standing. Orthostatic hypotension is significantly positively associated with falls in older adults and increased morbidity and mortality [16,17].

Clinical audits and feedback are common quality improvement strategies that offer clinicians an opportunity to measure how clinical care is provided and to assess whether set clinical standards are being met [18]. Audits are a tool for identifying areas of clinical care that need improvement, thereby focusing on education, research and quality improvement strategies to improve patient care and outcomes [19]. They constitute one of the pillars of clinical governance and can be described by the "plan", "do", "study", "act" phases that characterise the audit cycle.

In this study, we hypothesized that the percentage of elderly patients admitted with a hip fracture following a fall who had their LSBP measurements assessed while on admission as outlined in NICE clinical guidelines [4] could be improved upon by clinical audits, staff education, and by the effective use of aide-memoires and clinical proformas.

## Materials and methods

Ethical approval was given by the Salisbury District Hospital NHS Ethics Committee (CA_2020/21/4372).

A prospective study of patients above the age of 60 years admitted with a hip fracture following a fall between April 14 and May 25, 2020, in the Orthopaedics department was carried out. Fifty (50) patients (76% females) were included in this audit. The average age of patients was 83.4 years (SD = 8.03). The average length of hospital stay was 10 days. The electronic online observation charts (POET) and patients' notes while on admission were reviewed regularly for LSBP measurements recorded. Patients' notes were requested from the records department after discharge for further review.

The NICE guidelines for managing falls in older people were the clinical standards against which this audit was tested [4]. The results of this audit were presented at the mortality meeting of the Geriatric medicine department, and after brainstorming sessions, the following action plan was agreed upon: 

To increase awareness about OH on the wards using flyers and posters within the wards; To hold teaching sessions for ward staff on the standard procedure for measuring LSBP and immediately record this data onto the electronic database (POET) system; Re-assess compliance in three months.

**Figure 1:**
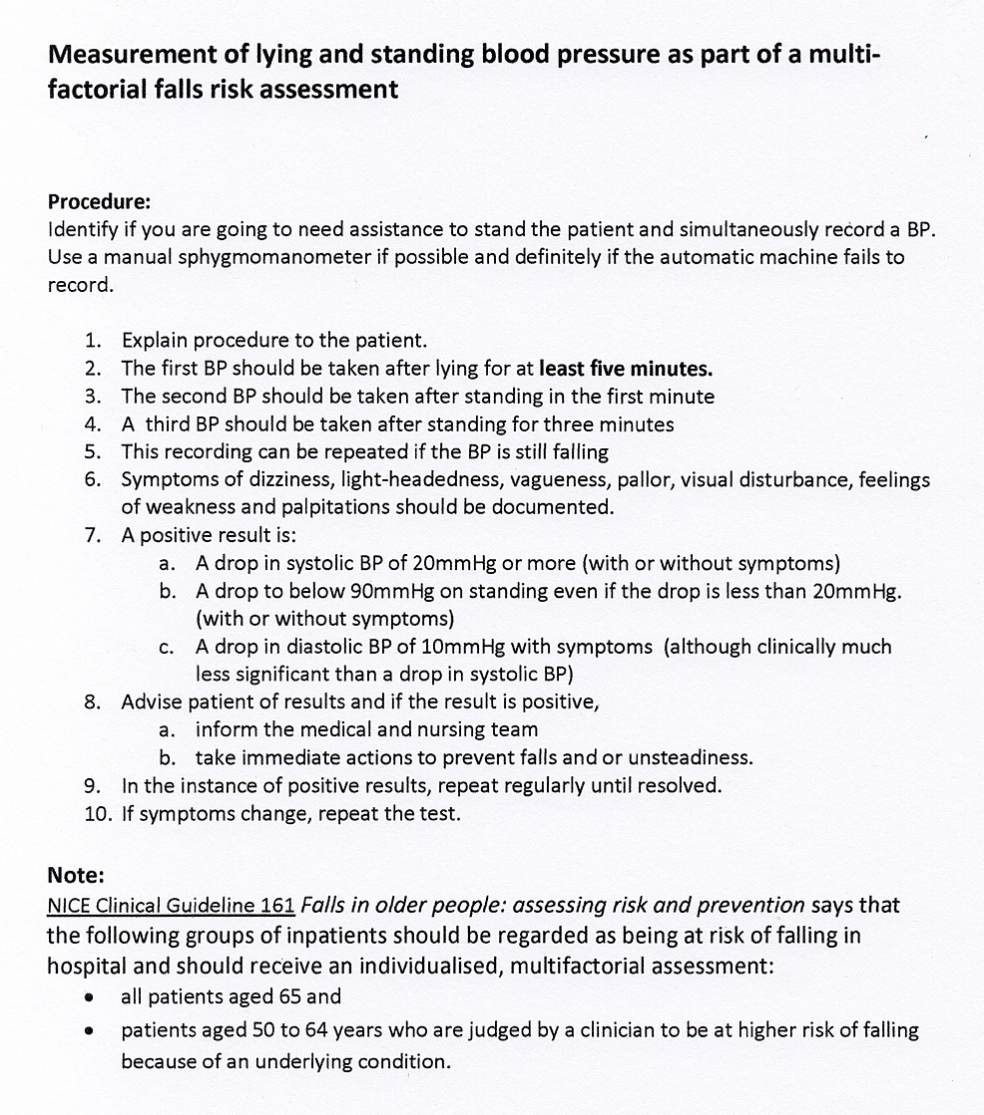
Measurement of orthostatic blood pressure. [20]

**Figure 2:**
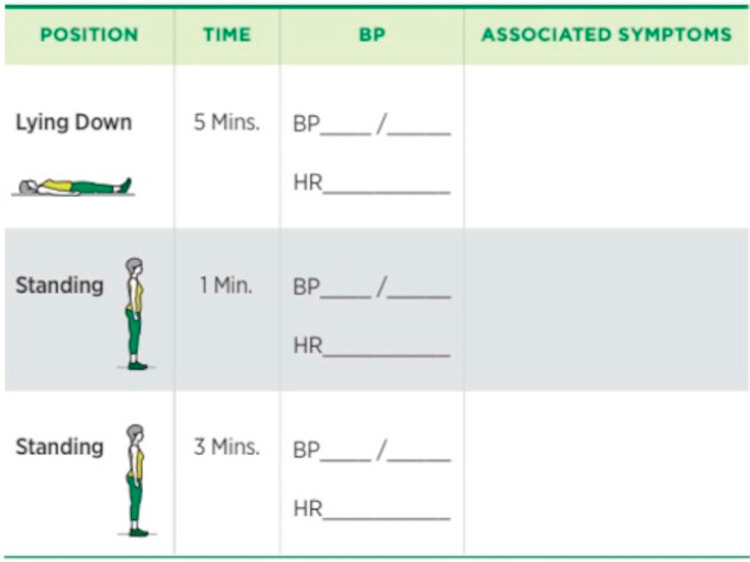
Documenting orthostatic blood pressure measurements [21]

Teaching sessions on the standard procedure for the measurement of LSBP as outlined in the Royal College of Physician's (RCP) Falls and Fragility Fracture Audit Programme (FFAP) 2017 guidance [6] were organized for all ward staff. Aide-memoires detailing the standard procedure for LSBP measurement and recording (Figures 1, 2) were placed in strategic locations around the wards.

A retrospective audit of patients above the age of 60, admitted with a hip fracture secondary to a fall between August 10 and September 21, 2020, in the Orthopaedics department was undertaken to assess progress and compliance with set standards. Fifty-eight (58) patients (73% females) were included in this re-audit. The average age of patients was 81.4 years (SD = 7.05), and the average length of hospital stay was 11 days. All statistical analysis was performed using SPSS (version 25.0; IBM Corp., Armonk, NY, USA). The results of each audit were compared and statistically analyzed using the student's t-test to compare continuous variables and the chi-square test for discrete variables. A p-value of <0.05 was considered statistically significant.

## Results

A total of 108 patients were reviewed over the two audit cycles. The common denominator was >60 years of age and admission to the Orthopaedics department following a fall. After the first audit, and following a root cause analysis, it was discovered that there was a lack of sufficient training amongst ward staff on the standard procedure for the accurate measurement and documentation of LSBP which led to poor compliance with set standards over time. Following the implementation of the action plan, the second audit showed a statistically significant improvement in both measurement and accurate documentation of LSBP values (Table 1). 

**Table 1:** Comparison of audit outcomes showing improvement *Chi-square, **T-test, ***Fischer exact test LSBP: lying and standing blood pressure

	First Audit (n=50)	Second Audit (n=58)	p value
Gender m:f	1:3.16	1:2.62	0.6715*
Mean age (yrs)	82.45±6.9	81.4±7.05	0.4375**
LSBP measured	16(32%)	45 (83.3%)	<0.0001*
LSBP correctly documented	12(75%)	45(100%)	0.0035***

## Discussion

This study attempted to show that clinical audit and implementation of staff education can be used as quality improvement tools for the accurate measurement and recording of LSBP in patients that are at high risk for falls. 

A falls risk assessment consists of a falls history, medication review, physical examination, functional, and environmental assessments. Because the risk of falls increases with the presence of more risk factors, risk can be significantly reduced by modifying some of these factors. Longitudinal studies have shown a consistently positive association between OH and falls and have found OH to be causal rather than a consequence of falls [16]. OH, when symptomatic, can present as a feeling of light-headedness, dizziness, fatigue, altered mentation, blurred vision or syncope when standing from a recumbent position. It is the most common modifiable risk factor for falls in older people [22]. Causes of OH include drugs (anti-hypertensives, diuretics, peripheral vasodilators etc.), anaemia, sepsis, autonomic failure, carotid sinus disease and prolonged bed rest following an illness. Because not all patients who have OH are symptomatic, it should be searched for repeatedly in high-risk patients. The NICE Clinical Guideline for Falls in older people recommends that the following groups of inpatients should be regarded as being at risk of falling in hospital and should receive an individualized, multifactorial assessment: all patients aged 65, and patients aged 50 to 64 years who a clinician judges to be at higher risk of falling because of an underlying condition. 

Many patients who meet these criteria do not get their LSBP measurements correctly taken and recorded as indicated by the RCP, FFAP 2019-2021 document [20]. Even when done, there are inconsistencies in the procedural sequence and specific timing of BP measurements by ward staff. This can be attributed to a lack of proper clinical training on the practicalities involved in LSBP measurements, and a lack of awareness of set guidelines. In the first audit cycle, 68% of patients on the ward who were above the age of 60 and presented with a hip fracture following a fall were not assessed for OH. When LSBP was measured, it was wrongly recorded 25% of the time. Measuring LSBP is a complex procedure that is often impractical as patients are required to have been erect for a minimum of three minutes before erect blood pressure measurements are taken. Our root cause analysis after the first audit cycle identified poor staff training and the need for assistance when measuring LSBP (most patients require assistance of at least one person to stand erect post-operatively), as the main cause of non-compliance with set standards. Being conscious of the Hawthorne effect, where a subject alters their behaviour due to their awareness of being observed, teaching sessions explaining the physiology and pathology of OH, coupled with instituting a system of constant feedback and reminders across all orthopaedic wards. The results of this were significant improvements in LSBP measurement and recording by ward staff by the next audit cycle. 

Other strategies for falls prevention in the elderly are: an MDT approach to care, interventions that increase strength and balance such as the Otago exercise programme and community group classes, collaborating with patients and carers to effect behavioural change, reducing the chances of a fall injury by improving and optimising bone health through the use of calcium and vitamin D supplements, and the use of personal medical alerts in high-risk patients to prevent a long lie in the event of a fall [23,24].

There are several limitations of this study. We focused on the measurement or not of LSBP, hence, data on exact blood pressure recordings were not considered or analysed. Also, due to the variability in patients' post-operative recovery, the exact timing of LSBP measurement was not considered as some patients require more time to be mobilised when compared to others. Patients’ records were however assessed throughout the duration of their in-hospital stay for LSBP recordings. Furthermore, improvements in LSBP measurement in high-risk patients recorded after the first audit may not be sustainable in the long term without constant staff education and feedback.

## Conclusions

Orthostatic hypotension is common in hospitalized elderly patients and is a significant cause of falls in this age group. In this study, we showed that accurate measurements and recording of LSBP by ward staff improved significantly with teaching sessions and the use of clinical proformas thereby confirming audits to be a strong tool in clinical quality improvement. However, sustenance of these gains requires routine staff education and implementation of regular audit.
